# Mouth Wash for Tobacco Consumers

**Published:** 1881-12

**Authors:** 


### MOUTH WASH FOR TOBACCO CONSUMERS.

C. Graham, M. D., Chicago, writes: Bromo-chloralum, twenty to thirty drops, in a tablespoonful of water, forms an excellent deodorizing mouth wash, where it becomes desirable to at once destroy the effect upon the breath of tobacco smoking or chewing. It acts like a charm—it being odorless itself, yet destroying instantly the after-effect of the weed upon the breath.—*Amer. Specialist.*

